# Contribution of Each Tryptophan to the Total Fluorescence Emitted by a Protein with Multiple Tryptophan Residues Depends on the Energy of the Excitation Radiation Quantum

**DOI:** 10.1021/acsomega.4c08874

**Published:** 2024-10-16

**Authors:** Karolina Stachurska, Jan M. Antosiewicz

**Affiliations:** Biophysics Division, Institute of Experimental Physics, Faculty of Physics, University of Warsaw, Pasteura 5 St., 02-093 Warsaw, Poland

## Abstract

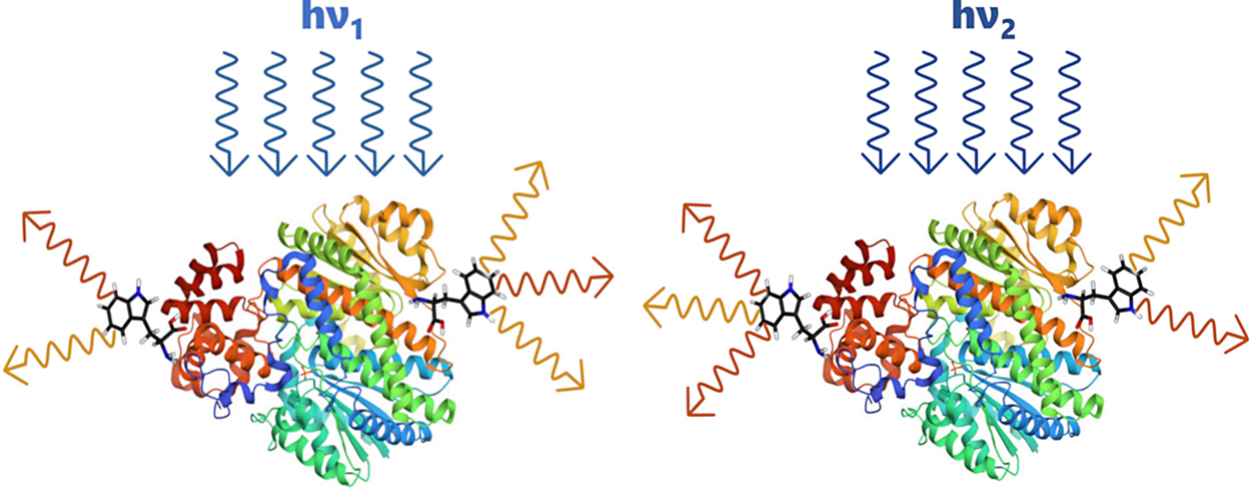

The structural changes induced by the addition of sodium dodecyl sulfate (SDS) in chymotrypsin and chymotrypsinogen were studied by the stopped-flow kinetic method with tryptophan fluorescence observation of the transients. Four fluorescence excitation wavelengths were used: 222, 260, 280, and 295 nm. It was found that the recorded transients were dependent on the excitation wavelength. The difference emission spectra between the complex and the free enzyme recorded for different excitation wavelengths are different. They contain a positive limb of fluorescence enhancement around 380 nm and a limb of fluorescence quenching around 340 nm. Their relative sizes depend on the excitation length, which fully explains the kinetic observations and proves that the contribution of tryptophans distributed at different sites within the protein molecule to its total fluorescence depends on the excitation wavelength. This is an important and novel finding that goes beyond the well-known fact that tryptophans distributed at different sites in the protein molecule have different fluorescence intensities.

## 1 Introduction

In our recent work we have investigated the kinetics of structural changes in α-chymotrypsin after mixing its solutions with sodium dodecyl sulfate (SDS) in a stopped-flow spectrometer with simultaneous recording of circular dichroism (CD) and fluorescence signals.^1^ Because of the changes that occur in the CD spectra of chymotrypsin under the influence of SDS, we carried out kinetic studies at excitations of 222 and 260 nm. The most interesting result of our work was the finding that the kinetics of structural change of chymotrypsin under the addition of SDS obtained from fluorescence measurements at the two excitations are different, although they both relate to the tertiary structure of the protein.

The intrinsic fluorescence of proteins originates from their aromatic amino acids, tryptophan, tyrosine and phenylalanine,^2^ even when excited by far-UV radiation.^3^ As we used a 320 nm cutoff filter (Schott WG 320) for the fluorescence measurements, the recorded fluorescence came from tryptophan residues,^4^ of which there are eight in chymotrypsin.

The interpretation of the intrinsic fluorescence of a protein with multiple tryptophan residues is complicated because it is not known a priori whether all Trp contribute equally to the total fluorescence emitted by the protein molecule.^5^ We interpreted the results of our kinetic studies with chymotrypsin by formulating the hypothesis that the contribution of individual Trps to total protein fluorescence varies and depends on the excitation wavelength.^1^

As a simple test of our hypothesis, we measured the steady-state fluorescence spectra of chymotrypsin in phosphate buffer and compared, after normalization, the spectra obtained with excitation at 222 and 260 nm. If the different Trp residues are excited differently, we expect different normalized equilibrium emission spectra. Indeed, the chymotrypsin spectra showed small but significant differences. On the other hand, the normalized spectra of free tryptophan in phosphate buffer recorded for both excitation wavelengths overlapped almost perfectly. Similar observations were made for solutions with the addition of SDS.

In this paper, we try to obtain further experimental evidence to confirm the validity of our hypothesis. We increased the number of selected excitation wavelengths (222, 260, 280, and 295 nm) and added chymotrypsinogen, a precursor of chymotrypsin, as a test system, but limited ourselves to fluorescence kinetic studies using the SX20 stopped-flow spectrometer (Applied Photophysics Ltd.). We also measured steady-state fluorescence spectra. Both reaction kinetics and steady-state spectra were recorded using a 320 nm cutoff filter (Schott WG 320).

The inclusion of chymotrypsinogen in our study proved to be particularly fortunate. The results of the stopped-flow experiments obtained for this protein reminded us of Halford’s excellent work describing the kinetics of the association of lysozyme with *N*-acetylchitotriose^6^ and allowed us to formulate the innovative hypothesis presented in the title of our paper.

## 2 Methods

### 2.1 Materials

Chemicals used in this study were purchased from Sigma-Aldrich or ROTH. Bovine pancreatic chymotrypsin (Sigma-Aldrich, essentially salt-free, lyophilized powder, Art. No. C4879), bovine pancreatic chymotrypsinogen (Sigma-Aldrich, essentially salt-free, lyophilized powder, Art. No. C4879), L-tryptophan (ROTH, ≥98.5%, Ph. Eur. for Biochemistry, Art. No. 4858.1), Sodium dodecyl sulfate (SDS, ROTH, ≥99%, for electrophoresis, for biochemistry and molecular biology, Art.-No. 2326.1), Sodium dihydrogen phosphate monohydrate (ROTH, ≥98%, Ph. Eur, ACS, Cat. No. K300.1), Disodium hydrogen phosphate dehydrate (ROTH, ≥98%, p.a., ACS, Cat. No. 4984.2), Sodium chloride (ROTH, ≥98%, p.a., ACS, ISO, Cat. No. 3957.1).

Chymotrypsin, chymotrypsinogen, SDS, and L-tryptophan were dissolved in sodium phosphate buffer (10 mM, pH = 7.0) prepared with ultrapure Millipore Milli-Q water (resistivity 18.2 MΩ·cm). The final solutions of proteins (10 μM) and tryptophan (80 μM) to be used in mixing experiments were prepared using known absorption coefficients ε_282nm_ = 51,000 M^–1^ cm^–1^, for chymotrypsin, ε_282nm_ = 51840 M^–1^ cm^–1^, for chymotrypsinogen, and ε_280nm_ = 5600 M^–1^ cm^–1^ for tryptophan. A stock solution of SDS (320 mM) was prepared by weight, and dilutions of this solution were prepared by appropriate mixing with the buffer.

### 2.2 Spectrophotometric Measurements

UV–vis absorption spectra were recorded with a UV-2401-PC spectrometer (Shimadzu). Fluorescence emission spectra were recorded using a Cary Eclipse Fluorescence Spectrophotometer (Agilent Technologies) in a 10 × 10 mm quartz cuvette. The excitation wavelength was set at either 222, 260, 280, or 295 nm. Spectra were recorded from 300 to 550 nm. To ensure measurement conditions consistent with the recording of fluorescence in our kinetic studies (see below), fluorescence spectra were collected through a 320 nm cutoff filter (Schott WG 320) used in the SX20 stopped-flow spectrofluorimeter. The widths of the emission and excitation slits were set to 5 nm, the detector voltage was 600 V, and the scan speed was 30 nm/min. The cell was thermostated using the Cary Single Cell Peltier Accessory (Agilent Technologies). An emission spectrum was recorded for each sample. All spectroscopic measurements were performed at 20 °C.

Fluorescence spectra were obtained from the following solutions: 5 μM protein solution in buffer, 5 μM protein solution in 20 mM and 40 mM SDS, 40 μM tryptophan solution in buffer, 40 μM tryptophan solution in 20 mM and 40 mM SDS, 20 and 40 mM SDS solutions in buffer, and buffer alone. Both chymotrypsin and chymotrypsinogen contain 8 tryptophan residues, so comparative tests with tryptophan solutions required a concentration of 40 μM.

Recorded fluorescence spectra for a given compound were normalized to a maximum of 100 units and a comparison was made between different excitation wavelengths.

### 2.3 Kinetic Experiments and Their Analysis

An SX20 spectrofluorimeter from Applied Photophysics Ltd. was used for the kinetic experiments. The emission was collected at 90 deg to the excitation beam using a 320 nm cutoff filter (Schott WG 320). The excitation path was 2 mm for all wavelengths. The emission path was 1 mm for all wavelengths. For each wavelength, the voltage of the fluorescence photomultiplier tube was adjusted to provide an output signal of 8 V after mixing 10 μM protein or 80 μM tryptophan solution with the phosphate buffer.

We performed two series of stopped-flow experiments differing in the initial concentration of SDS—80 and 40 mM. Both gave final concentrations above the CMC after mixing in the stopped-flow cell. For each pair of mixed solutions, 5 reaction progress curves recorded in the stopped-flow experiments were averaged. All kinetic measurements were performed at 20 °C.

For each excitation wavelength, the following mixing experiments were performed: buffer–buffer, protein/tryptophan-buffer, SDS-buffer, protein/tryptophan-SDS.

The final protein or tryptophan concentrations after mixing were the same as in our steady-state fluorescence spectra measurements.

The fluorescence reaction progress curves were analyzed using the program DynaFit 4 by Kuzmič.^7,8^ Since the results of the numerical analysis of the reaction progress curves reflecting the structural changes of the studied proteins under the influence of the addition of SDS are not crucial for the formulation of the conclusions presented in this paper, the methodological details of this analysis and the obtained results are presented in the Supporting Information (SI) file.

## 3 Results and Discussion

**Figure 1:**
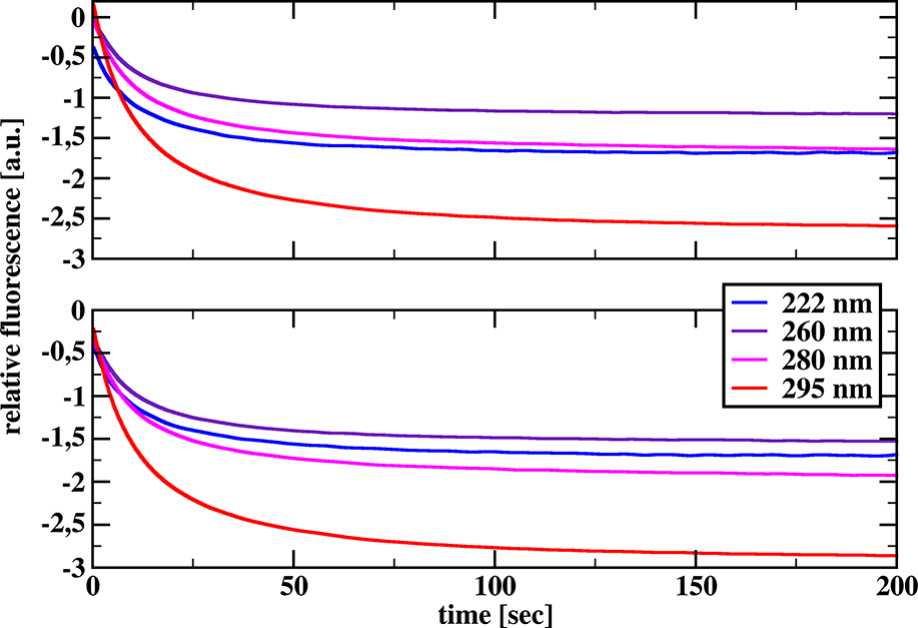
Relative tryptophan fluorescence transients recorded after mixing 10 μM solution of α-chymotrypsin with 40 (top) and 80 (bottom) mM solution of SDS for different excitation wavelengths.

**Figure 2:**
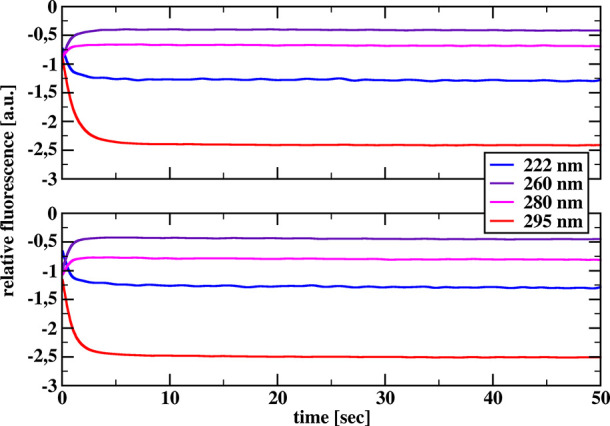
Relative tryptophan fluorescence transients recorded after mixing 10 μM solution of chymotrypsinogen with 40 (top) and 80 (bottom) mM solution of SDS for different excitation wavelengths.

Figures 1 and 2 show the relative reaction progress curves recorded for chymotrypsin and chymotrypsinogen, respectively, after mixing a 10 μM protein solution with a 40 (upper graphs) or 80 (lower graphs) mM SDS solution for all four excitation wavelengths used.

The relative reaction progress curves shown represent the differences between the sum of the curves recorded for protein-SDS and buffer–buffer mixing and the sum of the curves recorded for protein-buffer and SDS-buffer mixing. Therefore, these relative reaction progress curves only show changes occurring in the protein molecule. The relative progress curves that would be obtained by subtracting the progress curve recorded after mixing the protein solution with the buffer from that obtained after mixing the protein solution with the SDS solution look very similar, but contain small, practically negligible contributions from the surfactant.

All relative reaction progress curves obtained for chymotrypsin and chymotrypsinogen were analyzed by fitting a model described by the sum of two exponential terms and a linear term given by eq S1 in the SI file. The results of these fits are presented in Supplementary Table S1 in the SI file. In general, the results presented in Supplementary Table S1 confirm that the relaxation times characterizing the kinetics of the ongoing transformations of the tertiary structures of chymotrypsin and chymotrypsinogen are different for different excitation wavelengths. As mentioned above, the protein concentrations used in the kinetic measurements were relatively low, and obtaining a very good signal-to-noise ratio in the recorded reaction progress curves would require a much larger number of mixing experiments for each pair of solutions in the stopped-flow spectrometer cell to obtain sufficiently accurate average progress curves. However, obtaining very accurate quantitative results was not our goal. We only wanted to document the quantitative differences between reaction progress curves recorded at different excitation radiation lengths. The results shown in Supplementary Table S1 are quite sufficient for that.

It is important to note these quantitative differences when considering the results obtained for chymotrypsin, because qualitatively all the relative reaction progress curves look similar. Namely, all these curves show fluorescence quenching over time. We can also emphasize that the reaction progress curves for chymotrypsin obtained at 222 and 260 nm excitations are fully consistent with the curves obtained in independent experiments described in our previous work.^1^

For chymotrypsinogen, we saw a very significant difference. Thus, the inclusion of chymotrypsinogen in our study proved to be particularly fortunate. Indeed, in the case of chymotrypsinogen, the change in the nature of the reaction progress curves at excitation wavelengths of 222 and 295 nm compared to excitation wavelengths of 260 and 280 nm is remarkable: at excitation wavelengths of 222 and 295 nm we see fluorescence quenching over time, whereas at excitation wavelengths of 260 and 280 nm we see an enhancement of chymotrypsinogen fluorescence over time.

As can be seen in Supplementary Table S1, the relaxation times characterizing the conformational transitions of chymotrypsinogen at 222 nm excitation differ from those at 295 nm excitation, and the same is true for 260 and 280 nm excitation. More important and interesting, however, is the qualitative change that becomes apparent when comparing the reaction progress curves at 222 and 295 nm excitation with those obtained at 260 and 280 nm excitation. These qualitative differences observed for chymotrypsinogen turned out to be particularly important because they reminded us of Halford’s excellent work describing the kinetics of the association of lysozyme with *N*-acetylchitotriose in a stopped-flow spectrometer with tryptophan fluorescence measurement excited by 290 nm radiation.^6^ Halford’s experiments were performed in three different buffers: citrate-citric acid (both 5 mM) for pH 4.4, 0.01 M-pyrophosphate adjusted to pH 5.9 with H_3_PO_4_, and 0.01 M-Tris adjusted to pH 7.4 with HCl. Most interesting for us are the measurements at pH 4.4 and 7.4, where the fluorescence observations were made after mixing the lysozyme and chitotriose solutions using the ‘band-pass filter’, which transmits light between 325 and 380 nm. Technically, therefore, the signal recording was quite similar to our experiments.

When the *N*-acetylchitotriose binding experiment to lysozyme was performed in a pH 4.4 buffer, Halford observed a quenching of the fluorescence recorded through the band-pass filter over time. However, in a pH 7.4 buffer, he observed an enhancement of fluorescence over time. To interpret these kinetic observations, Halford determined the difference emission spectra between the lysozyme-*N*-acetylchitotriose complex and the free enzyme under the conditions in which the kinetic experiments were performed. The difference spectra reflect both the wavelength and intensity perturbations caused by ligand binding and their variation with pH.^6^

The difference spectra obtained by Halford contain a positive limb of fluorescence enhancement around 320 nm and a limb of fluorescence quenching at or above 360 nm. Knowing that over 80% of lysozyme’s fluorescence comes from two of its six tryptophan residues, namely tryptophans 62 and 108, Halford presented and justified the hypothesis that the positive limb in the difference emission spectra between the complex and the free enzyme can be assigned predominantly to an enhancement in the fluorescence of tryptophan-108 of lysozyme and the negative limb to a quenching of tryptophan-62. Subsequently, Halford assigned the fluorescence quenching seen in the bimolecular step of *N*-acetylchitotriose binding at pH 4.4 to tryptophan-62 and the fluorescence enhancement seen on the subsequent rearrangement of the enzyme–ligand complex at pH 7.4 to tryptophan-108. From the perspective of our research, it is important that the total fluorescence of lysozyme recorded using a band-pass filter transmitting radiation in the range of 325–380 nm, at pH 4.4, is dominated by tryptophan 62, and at pH 7.4 by tryptophan-108. This is an indication that the contributions of the different tryptophans to the total emitted fluorescence may be different for different solvent conditions.

We measured the steady-state fluorescence spectra of solutions of chymotrypsin and chymotrypsinogen in phosphate buffer without and with the addition of SDS and constructed difference spectra analogous to those presented by Halford.

**Figure 3:**
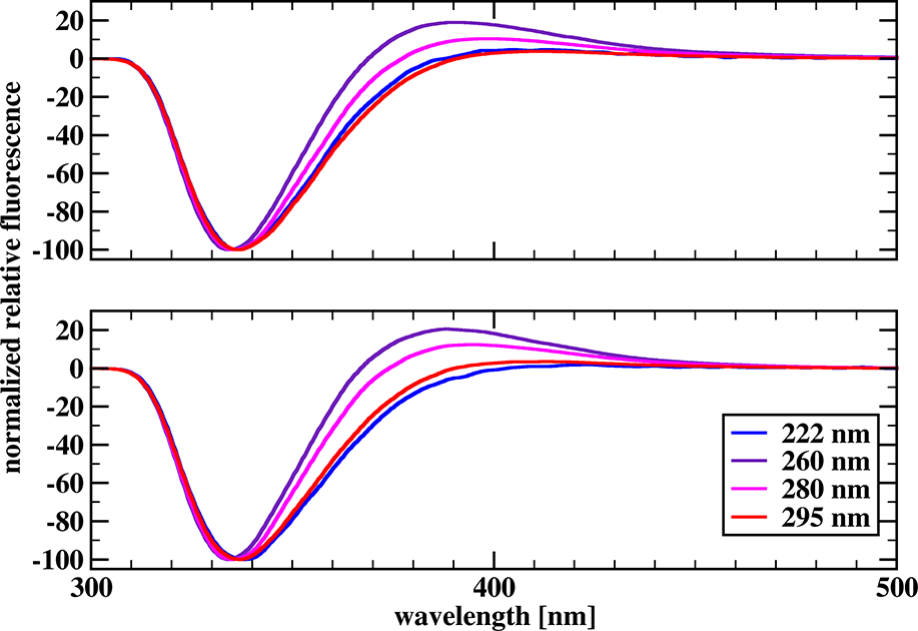
Normalized relative fluorescence spectra obtained by subtracting the fluorescence spectrum of a 5 μM solution of α-chymotrypsin in the phosphate buffer from the spectrum of a 5 μM solution of α-chymotrypsin in the presence of 20 mM (top) and 40 mM (bottom) SDS for different excitation radiation lengths.

**Figure 4:**
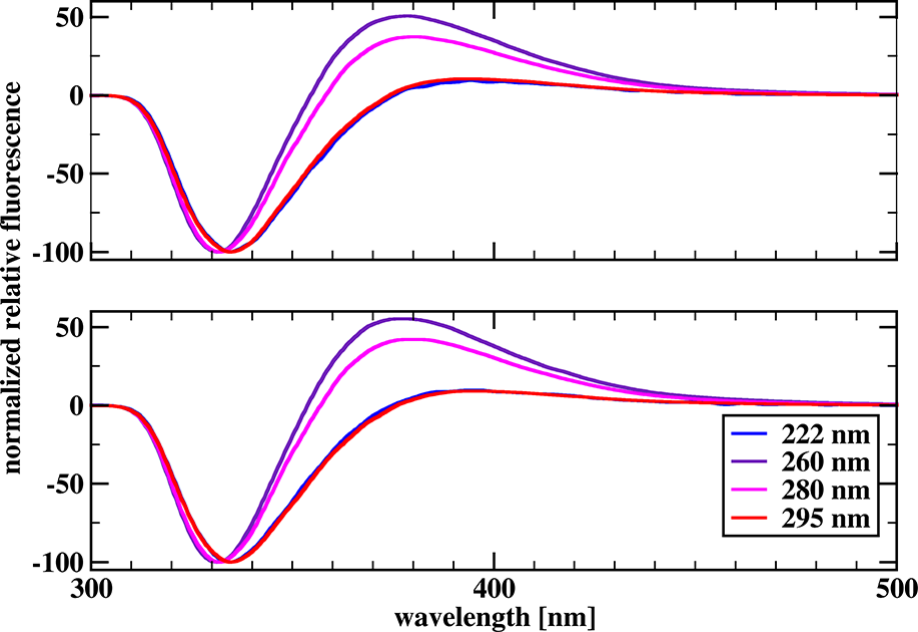
Normalized relative fluorescence spectra obtained by subtracting the fluorescence spectrum of a 5 μM chymotrypsinogen solution in the phosphate buffer from the spectrum of a 5 μM chymotrypsinogen solution in the presence of 20 mM (top) and 40 mM (bottom) SDS for different excitation radiation lengths.

Figures 3 and 4 show the relative steady-state spectra for chymotrypsin and chymotrypsinogen, respectively, in the 300–500 nm range, for two concentrations of SDS, and for all four excitation wavelengths, normalized to −100 units in the minima at a wavelength of about 340 nm. Our difference spectra also contain a positive limb of fluorescence enhancement around 380 nm and a limb of fluorescence quenching around 340 nm. Normalizing the difference spectra to −100 units in the minima allows better insight into the changes occurring in the positive limb.

It is important to realize that the fluorescence measured in a stopped-flow experiment using a band-pass filter (as in Halford’s case) or a cutoff filter (as in our case) at each instant corresponds to the integral under the curve representing the fluorescence spectrum of the solution under investigation. If the area under the fluorescence band increases, we will observe an increase in fluorescence in the reaction progress curve; if it decreases, we will observe a decrease in fluorescence over time. The spectra shown in Figures 3 and 4 are steady-state difference spectra and reflect the situation after the system has reached equilibrium. The area under the difference spectrum tells us what the net effect is in terms of the change in the signal measured in the spectrometer relative to the value at time *t* = 0.

If the positive limb dominates, the integral fluorescence measured in the stopped-flow experiment increases over time. If the negative arm dominates, the integral fluorescence measured in the experiment will decrease over time.

In the case of proteins containing more than one or many tryptophans, some of them may increase fluorescence due to a process involving the protein, and some may decrease fluorescence due to this process. The net result for the fluorescence measured with a band-pass or cutoff filter depends on which dominates. Halford showed that he could change the sign of the integral fluorescence changes in a stopped-flow experiment by changing the pH of the solution. In our work, this is achieved by changing the fluorescence excitation length. This is the undeniable novelty of our work, which allows us to hypothesize that the probability of fluorescence photon emission from a given tryptophan in a protein depends on the energy of the excitation quantum. As in Halford’s work, this probability depended on the pH of the buffer used.

It is interesting to note that Halford’s analysis of stopped-flow experiments is unprecedented. Despite an extensive search of the SCOPUS database, we were unable to find a similar analysis. Only our approach is analogous to Halford’s, but we emphasize again that we do not change the solution conditions, but the excitation length.

In Halford’s research, the ratio of the size of the positive limb to the size of the negative limb depended on the pH of the buffer in which the experiment to bind *N*-acetylchitotriose to lysozyme was performed. In our research, this ratio depends on the length of the excitation radiation. Thus, our difference spectra reflect both the wavelength and intensity perturbations caused by SDS binding and their variation with excitation wavelength. This is a novel observation without precedent in the scientific literature.

For both chymotrypsin and chymotrypsinogen, the size of the positive limb is largest at 260 nm excitation and slightly smaller at 280 nm excitation. For 222 and 295 nm excitation, the positive limb sizes are comparable and significantly smaller than in the previous two cases. In the case of chymotrypsinogen for 260 and 280 nm excitation, the size of the positive limb is sufficient for the total fluorescence emitted above 320 nm to increase with time. In the remaining cases studied, the negative limb dominates and the reaction progression curves show a decrease in total fluorescence emitted above 320 nm with time.

If we consider the reason for the pH dependence of the difference spectra obtained by Halford, we should note that when the pH of the solution changes, the total electrical charge of the proteins changes, so at different pHs we are dealing with the formation of complexes by slightly different protein molecules. In our studies, we do not change the solution conditions, so each time we are dealing with the same process of changing protein structure under the influence of SDS. Therefore, the natural question is why the difference spectra obtained in our research depend on the length of the excitation radiation. And the title of our work provides a reliable answer.

Halford was able to identify the tryptophans responsible for the observed changes on the basis of previously published work. This enabled him to understand the molecular mechanisms involved in binding chitotriose to the active site of lysozyme. We do not have that possibility. However, that is irrelevant to our conclusion. What is known is this: there are tryptophans that increase fluorescence in the process tested, and there are tryptophans that decrease fluorescence in the process tested. Since the net result of increasing or decreasing integral fluorescence depends on the excitation length, it can be concluded that the probability of photon emission by each of the tryptophans present in the protein depends on this length. Knowing which tryptophans in chymotrypsinogen or chymotrypsin increase the number of fluorescence photons emitted during the conformational change of the protein and which decrease the number of photons emitted is completely irrelevant for assessing the credibility of our hypothesis.

In our previous work,^1^ we hypothesized that the contribution of each tryptophan to the total fluorescence emitted by a protein with multiple tryptophan residues depends on the energy of the excitation radiation quantum, but this hypothesis was based on the observation of relatively small differences between the relaxation times and amplitudes characterizing fluorescence quenching in recorded reaction progress curves. As a simple test of this hypothesis, we proposed to compare the normalized fluorescence spectra of chymotrypsin and free tryptophans in the presence and absence of SDS. We performed these measurements for both proteins studied in this work and for four excitation wavelengths. The results confirm our previous results, but we now believe that they are not as important in confirming our hypothesis as the difference spectra, the analysis of which was suggested by Halford. We also believe that it is worth leaving a trace of these measurements in this paper, but due to the above conclusions we are transferring the full description of their results to the Supporting Information file.

We also note that our hypothesis should not be surprising in principle, although to our knowledge no one has proposed it. This is probably due to the fact that fluorescence studies of proteins have been performed at excitations associated with long-wavelength absorption of aromatic chromophores, and no one has performed kinetic studies such as our recent work.^1^ However, the existence of the dependence mentioned in our hypothesis can be predicted on the basis of considerations based on molecular quantum mechanics related to the matrix method used to calculate circular dichroism spectra of proteins.^9−11^

In the matrix method, the protein is decomposed into *M* independent chromophores, with a monomer wave function ϕ_*is*_ for each chromophoric group *i* and electronic state *s*. The *k*th excited-state wave function of the protein, ψ^*k*^, is then written as a linear combination of electronic configurations, Φ_*ia*_, where only one chromophoric group, *i*, is in an excited state *a* and the others are in the ground state, 0^11^:
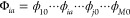
1where ϕ_*ia*_ is the wave function of chromophore *i* after the transition 0 → *a*. The excited state of a protein is described by the function
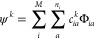
2The list of protein’s chromophores includes all those that can absorb radiation quanta in the range used in the study. In the case of the present work, we are interested in the 200–300 nm range. Proteins absorb ultraviolet radiation through the side groups of their aromatic amino acids, but in the far ultraviolet range an additional absorbing chromophore is the peptide bonds.^12^

If, as a result of absorbing a quantum of excitation radiation, the protein molecule moves to the state symbolically described by the eq 2, and finally a quantum of fluorescence is emitted by one of the tryptophans present in the protein molecule, there must be some way to dissipate the excess energy in a conversion to thermal vibrational energy, followed, if the originally excited chromophore is not tryptophan, by a possible resonance transfer of electronic energy to the lowest vibrational state of the singlet S1 electronic state of that tryptophan, from which a fluorescence quantum is emitted.^13^ Among the possible excitation energy transfers, those from tyrosine to tryptophan and from phenylalanine to tyrosine followed by tyrosine to tryptophan have been described.^14−17^ From this description, it is obvious that in the case of each quantum of excitation radiation absorbed by a protein molecule, both which of the chromophores will be excited and which of the tryptophans will emit a quantum of fluorescence are random events, although the corresponding quantum conditions imposed by the so-called “selection rules” must be fulfilled at the same time. Therefore, it should be expected that the probabilities of emission of a fluorescence quantum by each tryptophan will be different and dependent on the initial excitation energy. The problem of these probabilities is a problem of molecular quantum mechanics and a natural way to solve it would be to solve the appropriate time-dependent Schrödinger equation, but this currently appears to be an impossible task.

Although it seems impossible at present to prove the validity of our hypothesis using quantum mechanical calculations, the considerations set out above are useful. If this type of analysis leads to predictions of the CD spectra of proteins, it is also generally correct in our case, although it is practically impossible to implement. We feel it is important to point out that quantum mechanical considerations do not falsify our hypothesis.

In addition, we would like to point out that it is possible to determine which tryptophans in our protein and others that we will study in the future are responsible for the increase in fluorescence and which are responsible for the decrease in fluorescence by mutating selected tryptophans to other amino acids. However, that is a separate research program. And it should start with the study of ditryptophan proteins.

We would like to conclude this section by discussing the novelty of what we observed and found. We do this because a shorter version of this work submitted to another journal was rejected based on the opinion of the sole reviewer who felt that our work was a description of the phenomenon known as REES and was not novel. In the original manuscript we referred to the REES phenomenon and cited the work of Kwok et al.^18^ to conclude that our discovery had nothing to do with this phenomenon. This proved insufficient, so we present our arguments in more detail.

While searching the literature for publications describing fluorescence experiments in which the excitation wavelength was changed, we came across, among others, a review article by Demchenko,^19^ in which we read: “In 1970, three laboratories independently made a discovery that, for aromatic fluorophores embedded into different rigid and highly viscous media, the spectroscopic properties do not conform to classical rules. The fluorescence spectra can depend on excitation wavelength, and the excited-state energy transfer, if present, fails at the ‘red’ excitation edge existence of excited-state distribution of fluorophores on their interaction energy with the environment and the slow rate of dielectric relaxation of this environment.” These effects began to be referred to by the abbreviation REE, derived from the phrase “red-edge effects” that appeared in the above quote.

As far as we have been able to determine, the term “red-edge excitation shift” was first used by Lakowicz et al.,^20^ but without the abbreviation REES. However, the abbreviation REES appeared for the first time ten years after Lakowicz’s work, in the publication of Chattopadhyay and Mukherjee.^21^ The latter authors wrote: “REES serves as an indicator of the fluorophore environment”. In general, REES is defined as the shift in the wavelength of maximum fluorescence emission toward higher wavelengths, caused by a shift in the excitation wavelength toward the red edge of the absorption spectrum.

To the best of our knowledge, the first report of studies with changing the length of the excitation radiation while observing tryptophan fluorescence was reported by Demchenko and Ladokhin,^22^ although without giving it the name REES. To demonstrate that our observations of tryptophan fluorescence have nothing to do with the REES phenomenon, we refer to the work of Kwok et al.^18^ where we find normalized fluorescence spectra of free tryptophan solutions excited in the range of 292–310 nm. Of the excitation lengths we used, only 295 nm is in this range, but as you can see, the REES effect overlaps from 292 to 300 nm. For excitations at 300–310 nm, the long-term fluorescence spectra of free tryptophan are extended to several tens of nm for a normalized signal value below 10%, which is completely inconsistent with the image shown in our Supplementary Figure S5. Furthermore, it is obvious that the excitation lengths of 222, 260, and 280 nm do not belong to the red edge of the absorption spectrum of tryptophan.

## 4 Conclusions

We believe that the results of our experiments described above and the data presented in the SI file allow us to hypothesize that the intensity of fluorescence emitted by individual tryptophans in the polytryptophan protein depends on the primary energy of the excitation quanta, not because the fluorescence of the tryptophans per se depends on this energy, but because the paths of nonradiative loss of this primary energy toward the fundamental vibrational states of the lowest electronically excited singlet states of these tryptophans are realized with different probabilities for different energies of the primary excitation quanta. Therefore, our results have nothing to do with the phenomenon known as REES and are a real novelty in the literature on the subject.

## Supporting Information Available

Normalized fluorescence spectra for different excitation wavelength, long-wavelength parts of the normalized fluorescence spectra of 40 μM tryptophan at different solvent conditions, numerical analysis of the recorded fluorescence reaction progress curves, and locations of points corresponding to 50 units in the normalized fluorescence spectra (PDF)
