# Supplementary material for: Contribution of Each Tryptophan to the Total Fluorescence Emitted by a Protein with Multiple Tryptophan Residues Depends on the Energy of the Excitation Radiation Quantum
Source: ACS Omega. 2024 Oct 16;9(43):43998–4004. doi: 10.1021/acsomega.4c08874 (PMC11525737; doi:10.1021/acsomega.4c08874)
Supplement: Supplementary file 1 — ao4c08874_si_001.pdf [file ao4c08874_si_001.pdf]

Contribution of each tryptophan to the total fluorescence emitted by a protein with multiple tryptophan residues depends on the energy of the excitation radiation quantum

Karolina Stachurska and Jan M. Antosiewicz*

Division of Biophysics, Institute of Experimental Physics, Faculty of Physics, University of Warsaw, 02-093 Warsaw, Pasteura 5, Poland

E-mail: Jan.Antosiewicz@fuw.edu.pl

Phone: +48 22 55 32 340

Supporting Information

Numerical analysis of recorded reaction progress curves

The fluorescence reaction progress curves were analyzed assuming that they can be represented as the sum of a certain number of exponential terms. We used the program Dyna Fit 4 in the calculations.[1,2] The indications of the model discriminating algorithm, i.e. the most appropriate number of exponential terms, were not as clear as in the case of the previous project,[3] but this time we used a four times lower concentration of the protein (chymotrypsin or chymotrypsinogen), which slightly worsened the signal-to-noise ratio in the recorded reaction progress curves. The most frequently reported model was the sum of two exponential terms and a linear term describing the linear decrease of the signal with time, probably

caused by photobleaching of the fluorophores:

$$S(t) = A_0 + A_1 \cdot (1 - \exp(-k_1 t)) + A_2 \cdot (1 - \exp(-k_2 t)) - b \cdot t \tag{S1}$$

By introducing a linearly decreasing term into our model, we acted similarly to Otzen et al.[4] for β-sheet proteins. These authors fitted the kinetics of SDS-induced structural transitions to monoexponential functions with a linear drift, to account for photobleaching of Trp fluorophores. A similar approach was taken by Patel and colleagues, who analyzed the ATP turnover cycle of kinesins.[5]

The results of analyzing the recorded progress curves are presented in Table S1.

Normalized fluorescence spectra for different excitation wavelengths

We repeated the test performed in the previous works[3] for all four excitation wavelengths and both proteins and free tryptophan, which involved determining their normalized fluorescence spectra. The results are shown in Figures S1– S4 and in Table S2.

Our first step was to record the fluorescence spectra of solutions of free tryptophan and chymotrypsin and chymotrypsinogen in phosphate buffer, both with (20 or 40 mM) and without added SDS. The spectra were recorded at the four excitation wavelengths listed above, using a 320 nm cut-off filter to make the recording conditions as close as possible to the stopped-flow experiments. The concentrations of chymotrypsin and chymotrypsinogen in the fluorescence spectra measurements were 5 μM, and since they each contain eight tryptophan residues, the equivalent free tryptophan concentration was 40 μM. Comparison of the recorded spectra was performed after normalization to 100 units at the emission maximum.

In the case of normalized spectra recorded for free solutions of tryptophan molecules, we expect them to be identical for all excitation radiation lengths. Indeed, the two examples of such normalized spectra shown in Figure S1 show that they overlap almost perfectly.

The next two Figures, S2 and S3, show the normalized fluorescence spectra of 5 μM chymotrypsin and chymotrypsinogen solutions in phosphate buffer and in the same buffer with the addition of 20 mM SDS. The spectra of both proteins with the addition of 40 mM SDS are shown in Figure S4.

The conclusions are the same as in our prevoius work:[3] the normalized spectra of free tryptophan in phosphate buffer without and with added SDS overlap almost perfectly, while analogous spectra for the two proteins tested show small but repeatable differences in subsequent experiments.

Although the differences between the normalized fluorescence spectra of the protein solutions recorded for the different excitation wavelengths are small, there is no doubt that the differences shown cannot be interpreted as due to measurement inaccuracies, because the differences between the normalized spectra for free tryptophan shown in Figure S1 are smaller. All three groups of spectra are presented in the same wavelength range of emitted fluorescence (70 nm) and normalized fluorescence (60 units).

Of note is the quantitative analysis of the plots presented in Table S2. This table shows the wavelength values for which the normalized fluorescence value is 50 units. The original fluorescence spectra were recorded with a resolution of 0.46 nm. Smoothed and normalized spectra are also stored at this resolution, but projection onto the abscissa allows the wavelength at which the signal has a specific value to be determined with much higher resolution. Therefore, in Table S2, these abscissa values are given with three decimal places. As a quantitative measure of the non-overlap of the normalized fluorescence spectra of the tested solutions at the four excitation wavelengths used, we can take the greater of the values Δ_{left} and Δ_{right} for each of the tested solutions. In the case of free tryptophan solutions, we have Δ_{right}=0.327 for the TRP solution in buffer and Δ_{left}=0.250 for the TRP solution with the addition of 20 mM SDS. For the solutions of the tested proteins, the larger ones of the Δ_{left}, Δ_{right} pair range from 0.503 nm for the chymotrypsinogen solution with the addition of 20 mM SDS to 1.280 nm for the chymotrypsin solution in buffer. It confirms

that the differences in the normalized spectra of the solutions containing the proteins, although small, are actually larger than the differences in the spectra recorded for solutions containing equivalent amounts of free tryptophans. In the light of the hypotheses presented in the main article, it remains to be determined to what extent the small changes observed in the normalized spectra of protein solutions influence the reaction progress curves after mixing with SDS solutions at different excitation lengths. This undoubtedly requires further investigation. An obvious and relatively accessible avenue for such research is to mutate individual tryptophans, for example to phenylalanines, and study the consequences of such mutations.

Supplementary Figures

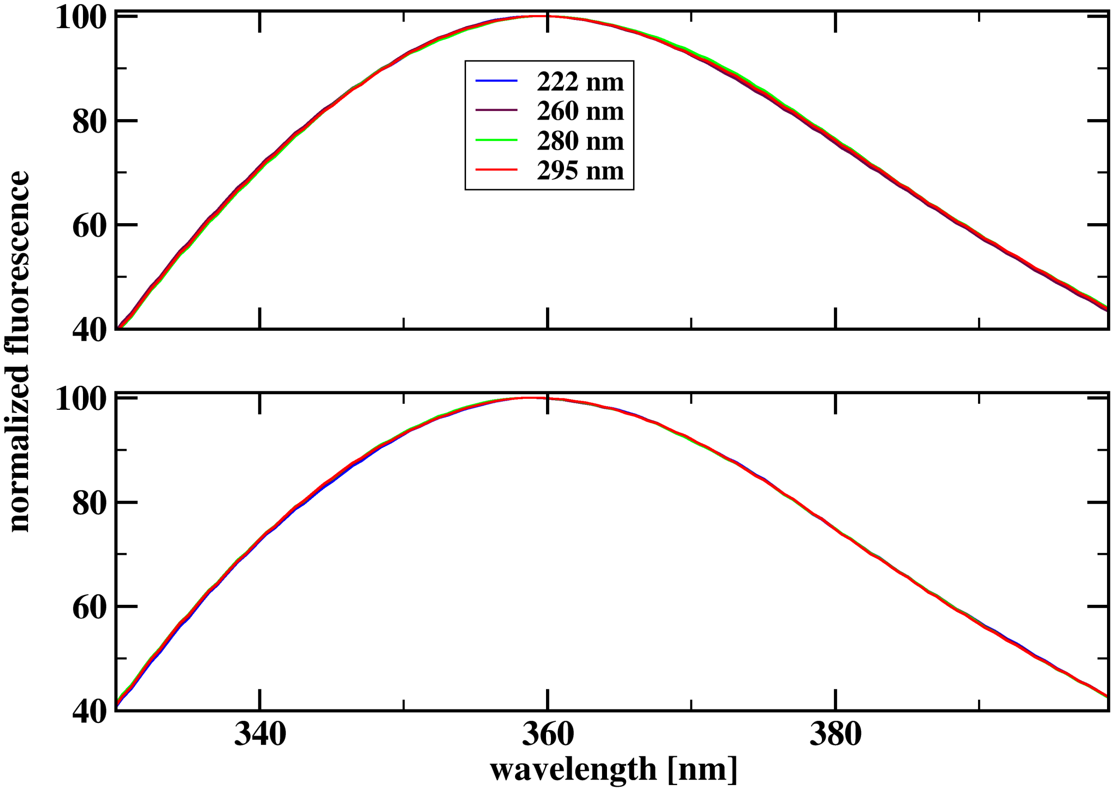

Figure S1: *TOP:* Normalized bufor corrected fluorescence spectra of 40 μM tryprophan solutions in phosphate buffer for different excitation wavelengths.; *BOTTOM:* Normalized bufor corrected fluorescence spectra of 40 μM tryprophan solutions in phosphate buffer in the presence of 20 mM SDS, for different excitation wavelengths. The spectra were recorded using a 320 nm cut-off filter (Schott WG 320)

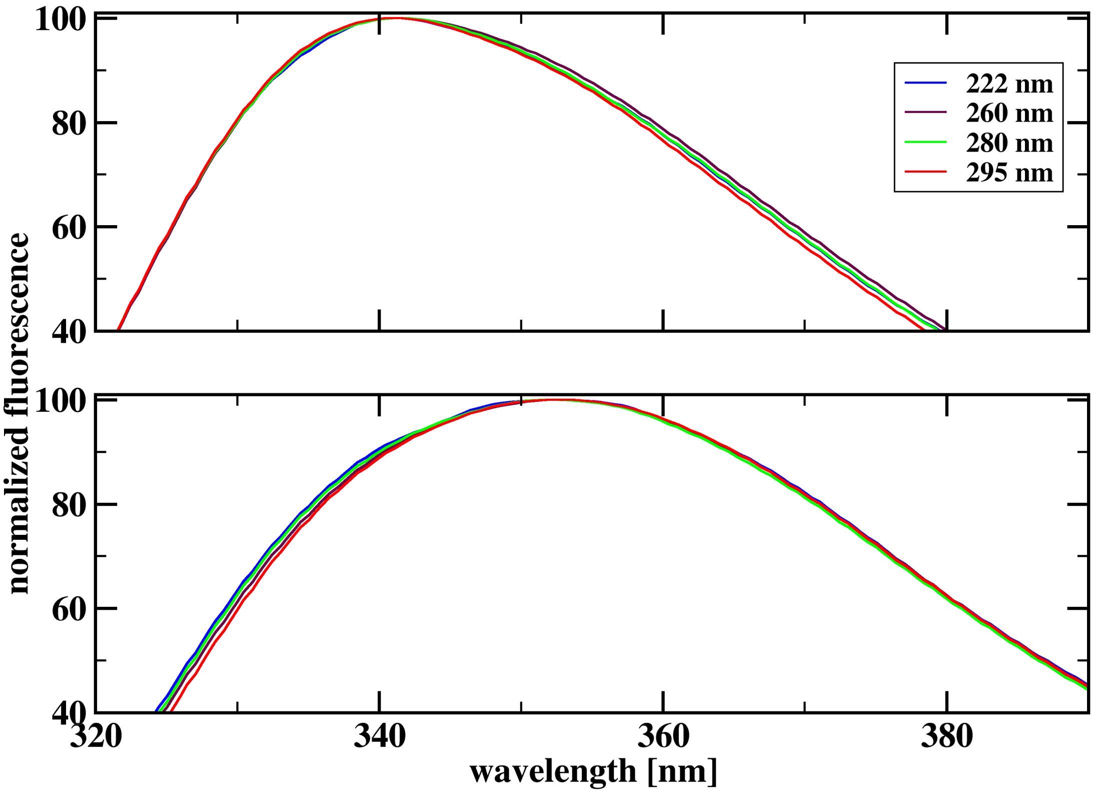

Figure S2: *TOP:* Normalized bufor corrected fluorescence spectra of 5 μM chymotrypsin solutions in phosphate buffer for different excitation wavelengths.; *BOTTOM:* Normalized bufor corrected fluorescence spectra of 5 μM chymotrypsin solutions in phosphate buffer in the presence of 20 mM SDS, for different excitation wavelengths. The spectra were recorded using a 320 nm cut-off filter (Schott WG 320)

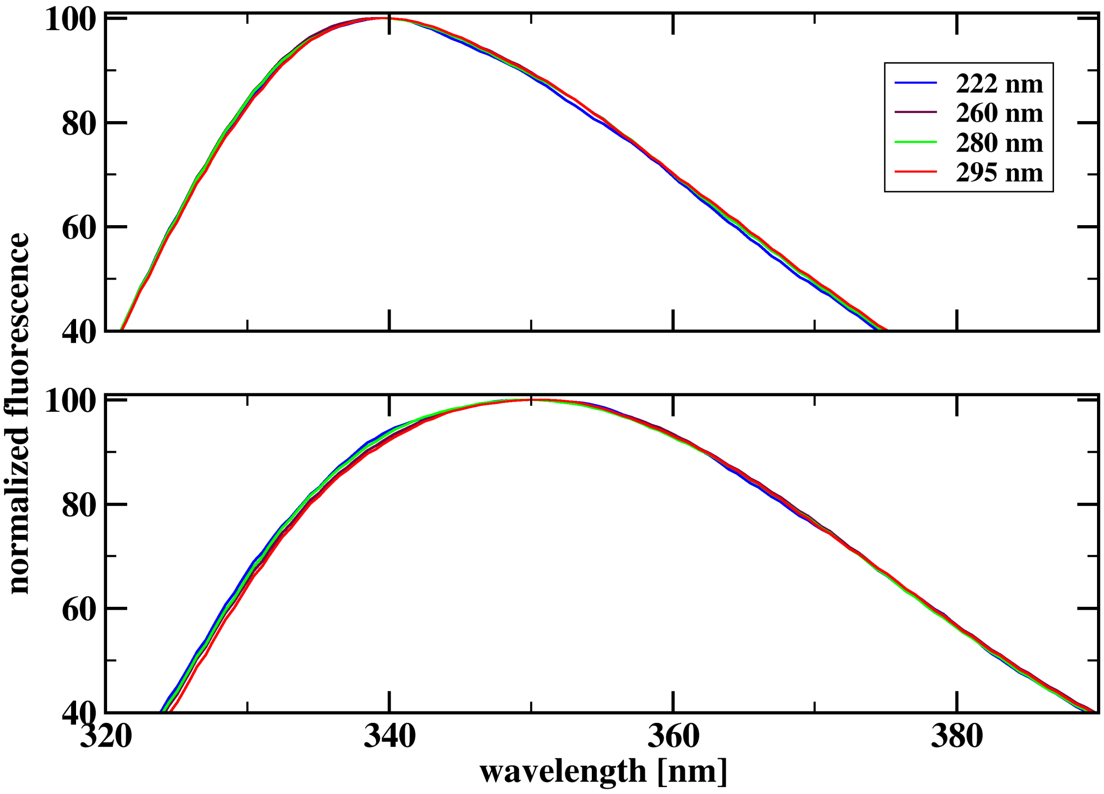

Figure S3: *TOP:* Normalized bufor corrected fluorescence spectra of 5 μM chymotrypsinogen solutions in phosphate buffer for different excitation wavelengths.; *BOTTOM:* Normalized bufor corrected fluorescence spectra of 5 μM chymotrypsinogen solutions in phosphate buffer in the presence of 20 mM SDS, for different excitation wavelengths. The spectra were recorded using a 320 nm cut-off filter (Schott WG 320)

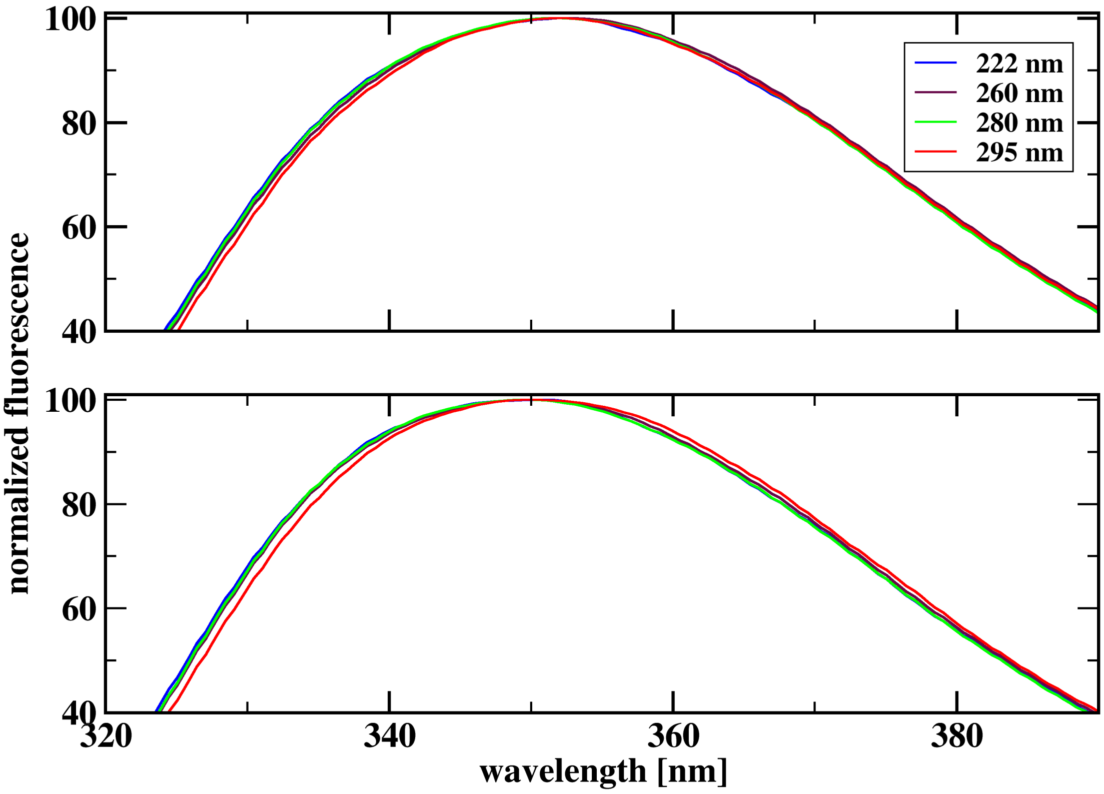

Figure S4: *TOP:* Normalized bufor corrected fluorescence spectra of 5 μM chymotrypsin solutions in phosphate buffer in the presence of 40 mM SDS, for different excitation wavelengths.; *BOTTOM:* Normalized bufor corrected fluorescence spectra of 5 μM chymotrypsinogen solutions in phosphate buffer in the presence of 40 mM SDS, for different excitation wavelengths. The spectra were recorded using a 320 nm cut-off filter (Schott WG 320)

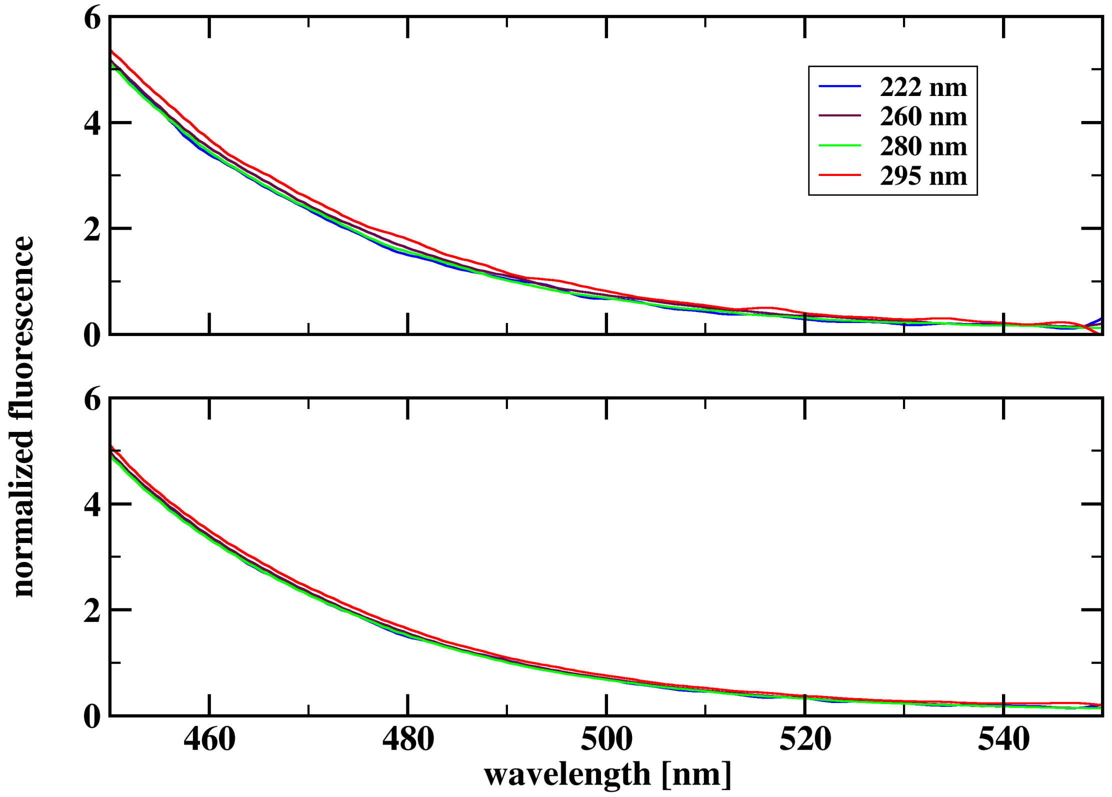

Figure S5: Long wavelength parts of the normalized fluorescence spectra of 40 μM tryptophan solutions in phosphate buffer, without addition (*TOP:*) and with addition of 20 mM SDS (*BOTTOM:*) at excitations 222, 260, 280 and 295 nm. The spectra were recorded using a 320 nm cut-off filter (Schott WG 320)

Supplementary Tables

Table S1: Results of the analysis with the DynaFit program,[1,2] fluorescence progress curves.

	protein concentation 5 μM			
	chymotrypsin		chymotrypsinogen	
	SDS 20 mM	SDS 40 mM	SDS 20 mM	SDS 40 mM
a0 222 nm	-0.397 ± 0.002	-0.362 ± 0.002	-0.573 ± 0.005	-0.664 ± 0.007
260 nm	-0.307 ± 0.001	-0.001 ± 0.001	-1.041 ± 0.002	-0.950 ± 0.002
280 nm	-0.301 ± 0.001	-0.002 ± 0.001	-1.104 ± 0.002	-0.902 ± 0.002
295 nm	-0.171 ± 0.001	0.000 ± 0.006	-1.044 ± 0.002	-0.805 ± 0.002
a1 222 nm	-0.696 ± 0.005	-0.645 ± 0.008	-0.560 ± 0.007	-0.385 ± 0.034
260 nm	-0.596 ± 0.003	-0.659 ± 0.003	0.623 ± 0.002	0.431 ± 0.009
280 nm	-0.813 ± 0.003	-0.786 ± 0.003	0.351 ± 0.002	0.180 ± 0.036
295 nm	-1.334 ± 0.004	-1.621 ± 0.036	-1.376 ± 0.002	-1.473 ± 0.006
k1 222 nm	0.158 ± 0.002	0.157 ± 0.002	2.04 ± 0.04	2.16 ± 0.17
260 nm	0.151 ± 0.001	0.141 ± 0.001	1.59 ± 0.01	1.82 ± 0.04
280 nm	0.146 ± 0.001	0.147 ± 0.001	1.63 ± 0.02	2.15 ± 0.24
295 nm	0.147 ± 0.001	0.100 ± 0.002	1.181 ± 0.004	0.979 ± 0.004
a2 222 nm	-0.566 ± 0.004	-0.647 ± 0.008	-0.119 ± 0.006	-0.210 ± 0.037
260 nm	-0.561 ± 0.003	-0.492 ± 0.003	-0.0510 ± 0.0019	0.127 ± 0.010
280 nm	-0.702 ± 0.003	-0.732 ± 0.003	-0.0298 ± 0.0014	0.061 ± 0.037
295 nm	-1.244 ± 0.004	-0.924 ± 0.024	-0.0742 ± 0.0016	-0.126 ± 0.006
k2 222 nm	0.0325 ± 0.0004	0.0365 ± 0.0005	0.322 ± 0.017	0.716 ± 0.064
260 nm	0.0399 ± 0.0002	0.0357 ± 0.0003	0.0236 ± 0.0019	0.562 ± 0.025
280 nm	0.0359 ± 0.0002	0.0372 ± 0.0002	0.171 ± 0.015	0.956 ± 0.217
295 nm	0.0347 ± 0.0001	0.0242 ± 0.0013	0.141 ± 0.007	0.262 ± 0.009
b 222 nm	0.00019 ± 0.00001	0.00018 ± 0.00001	0.00099 ± 0.00002	0.00068 ± 0.00002
260 nm	0.00033 ± 0.00001	0.00025 ± 0.00001	0.00000 ± 0.00005	0.00048 ± 0.00001
280 nm	0.00056 ± 0.00001	0.00059 ± 0.00001	0.00050 ± 0.00002	0.00053 ± 0.00001
295 nm	0.00058 ± 0.00001	0.00026 ± 0.00009	0.00033 ± 0.00003	0.00023 ± 0.00001

Table S2: The locations of points corresponding to 50 units in the normalized fluorescence spectra, and the range of their variability for the indicated excitation radiation lengths. All data are in [nm].

solution	λ_{exc}	$\lambda_{50,left}$	$\lambda_{50,right}$	Δ_{left}	Δ_{right}
CHA 5 μM	222	323.502	373.815	0.076	1.280
:	260	323.510	374.500		
buffer	280	323.435	373.912		
	295	323.434	373.220		
CHA 5 μM	222	326.630	387.063	1.002	0.615
:	260	327.211	386.964		
20 mM SDS	280	326.905	386.448		
	295	327.632	386.855		
CHA 5 μM	222	326.560	386.209	0.878	0.545
:	260	327.000	386.498		
40 mM SDS	280	326.771	385.953		
	295	327.438	386.189		
CHG 5 μM	222	322.571	369.431	0.138	0.519
:	260	322.435	368.912		
buffer	280	322.460	369.361		
	295	322.433	369.248		
CHG 5 μM	222	326.352	383.342	0.446	0.503
:	260	325.906	382.867		
20 mM SDS	280	325.950	382.839		
	295	326.073	383.309		
CHG 5 μM	222	325.769	383.330	1.001	0.696
:	260	326.070	383.519		
40 mM SDS	280	325.961	383.166		
	295	326.770	383.862		
TRO 40 μM	222	333.135	394.878	0.205	0.327
:	260	333.080	394.743		
buffer	280	333.285	395.070		
	295	333.207	395.016		
TRP 40 μM	222	332.668	394.282	0.250	0.197
:	260	332.505	394.188		
20 mM SDS	280	332.418	394.085		
	295	332.474	394.118		

References

(1) Kuzmic, P. Program DYNAFIT for the analysis of enzyme kinetic data: Application to HIV proteinase. *Anal. Biochem.* **1996**, *237*, 260–273, DOI:10.1006/abio.1996.0238.

(2) Kuzmic, P. DynaFit - A Software Package for Enzymology. *Meth. Enzymol.* **2009**, *467*, 247–280, DOI:10.1016/S0076-6879(09)67010-5.

(3) Stachurska, K.; Marcisz, U.; Dlugosz, M.; Antosiewicz, J. M. Kinetics of Structural Transitions Induced by Sodium Dodecyl Sulfate in α-Chymotrypsin. *ACS Omega* **2023**, *8*, 49137–49149.

(4) Nielsen, M. M.; Andersen, K. K.; Westh, P.; Otzen, D. E. Unfolding of β-Sheet Proteins in SDS. *Biophys. J.* **2007**, *92*, 3674–3685.

(5) Patel, J. T.; Belsham, H. R.; Rathbone, A. J.; Friel, C. T. Use of Stopped-Flow Fluorescence and Labeled Nucleotides to Analyze the ATP Turnover Cycle of Kinesins. *J. Vis. Exp.* **2014**, *92*, e52142/1–6.
